# To construct a risk prediction model for poor discharge readiness in patients undergoing elective unilateral lower extremity joint replacement based on clinical and psychosocial factors

**DOI:** 10.3389/fmed.2026.1811245

**Published:** 2026-07-22

**Authors:** Dan-dan Lin, Yu-cai Jiang, Lin-lin Zheng, Yao-ning Zhuang, Jian-huang Huang

**Affiliations:** 1 Department of Research, Affiliated Hospital of Putian University, Putian, Fujian, China; 2 Department of Pharmacy, Affiliated Hospital of Putian University, Putian, Fujian, China; 3 Department of Oncology, Affiliated Hospital of Putian University, Putian, Fujian, China; 4 Respiratory Critical Care Medicine Department, Affiliated Hospital of Putian University, Putian, Fujian, China; 5 Department of Neurosurgery, Affiliated Hospital of Putian University, Putian, Fujian, China

**Keywords:** discharge readiness, joint replacement surgery, machine learning, psychosocial factors, risk prediction model

## Abstract

**Objective:**

To construct and validate a machine learning model integrating clinical and psychosocial factors for predicting the risk of poor discharge preparation (PDP) in patients undergoing elective unilateral lower extremity joint replacement surgery.

**Methods:**

This study adopted a retrospective observational cohort design, enrolling 265 adult patients who underwent elective unilateral total hip or knee arthroplasty at a tertiary grade A hospital in Fujian Province from December 2022 to April 2023. Patients were divided into the PDP group and the well-prepared group based on the median total score of the Readiness for Hospital Discharge Scale (RHDS) (97.0 points). A total of 25 variables, including demographic, clinical, fear of movement (TSK-13), and socioeconomic factors, were collected. Variable selection was performed using strongly regularized Elastic Net logistic regression, and XGBoost and Elastic Net logistic regression models were constructed, respectively. Model performance was evaluated using 5 × 5 repeated stratified cross-validation, with metrics including AUC, PR-AUC, sensitivity, specificity, calibration, and decision curve analysis (DCA). The SHAP method was used to explain the prediction mechanism of the XGBoost model.

**Results:**

Elastic Net identified eight key predictors: length of hospital stay, postoperative kinesiophobia, monthly household income, educational level, preoperative kinesiophobia, age, and occupation. The XGBoost model performed the best, with an AUC of 0.833 (95% CI: 0.811–0.855) and a PR-AUC of 0.750 (95% CI: 0.708–0.790), significantly outperforming the logistic regression model (DeLong test *p* = 0.0035). The calibration curve showed good consistency, and DCA confirmed that it had the highest clinical net benefit within the threshold probability range of 5–40%. SHAP analysis indicated that the shortest length of hospital stay, high preoperative kinesiophobia level, and low household income were the three core risk drivers.

**Conclusion:**

This study successfully developed a high-precision and interpretable machine learning prediction tool that can effectively identify high-risk patients with poor discharge readiness. The model highlights the critical impact of shortening hospital stay, preoperative psychological state, and socioeconomic status on rehabilitation outcomes, providing a scientific basis for implementing early and personalized interventions.

## 1 Introduction

Elective unilateral lower extremity joint replacement surgery, as an important means of treating advanced joint diseases, has been widely applied in clinical practice, significantly improving the joint function and quality of life of patients (1). However, the postoperative rehabilitation process of patients is complex and varies among individuals, and the level of discharge readiness is directly related to the postoperative recovery effect and the risk of readmission (2). With the intensification of the aging population trend and the continuous increase in the number of patients undergoing elective lower extremity joint replacement surgery, how to effectively assess and enhance the discharge readiness level of patients has become an urgent issue to be addressed in the current clinical nursing and rehabilitation fields (3, 4).

Current research mainly focuses on the impact of postoperative physiological indicators and clinical pathological factors on patient recovery, while the role of psychosocial factors in patients’ discharge readiness and rehabilitation has increasingly drawn attention. Numerous studies have revealed that patients’ psychological states, such as kinesiophobia, anxiety and depression, as well as non-clinical factors like social support systems and economic conditions, significantly affect the smoothness of postoperative recovery and patients’ self-management abilities (5, 6). Moreover, patients with lower socioeconomic status often face insufficient access to medical resources and weak family support, which frequently leads to inadequate discharge preparation and increases the risk of postoperative adverse events. These findings suggest that relying solely on traditional clinical indicators is insufficient to comprehensively reflect patients’ discharge readiness, and integrating psychosocial factors is of great significance for constructing a precise risk assessment system (7, 8). Although the above studies have provided a theoretical basis for understanding patients’ discharge readiness, there is still a lack of systematic integration of clinical and psychosocial multi-dimensional factors to establish a predictive model for poor discharge readiness in the specific population of patients undergoing lower extremity joint replacement surgery (9). Most current risk assessment tools focus on a single dimension, making it difficult to capture the complex interactions among multiple factors, and lack the development and validation of models based on modern data analysis techniques such as machine learning (10). Therefore, how to utilize multivariate statistics and machine learning methods to construct efficient, accurate, and clinically interpretable predictive models for discharge readiness risk has become a hot and difficult issue in clinical research (11, 12).

To address the aforementioned deficiencies, this study adopted a retrospective observational cohort design to systematically collect clinical indicators, psychological status, and socio-economic background information of patients undergoing elective unilateral lower extremity joint replacement surgery. By integrating Elastic Net, logistic regression, and XGBoost algorithms, variable selection and model construction were carried out. This approach can effectively handle multicollinearity, variable selection, and nonlinear relationships, thereby enhancing the predictive performance and generalization ability of the model. The aim of this study is to develop a risk prediction tool for poor discharge readiness based on the combination of clinical and psychosocial factors, providing a scientific basis for the early identification of high-risk patients and the formulation of personalized intervention plans in clinical practice (13, 14).

## 2 Methods

### 2.1 Study design and sample size estimation

**Figure 1:**
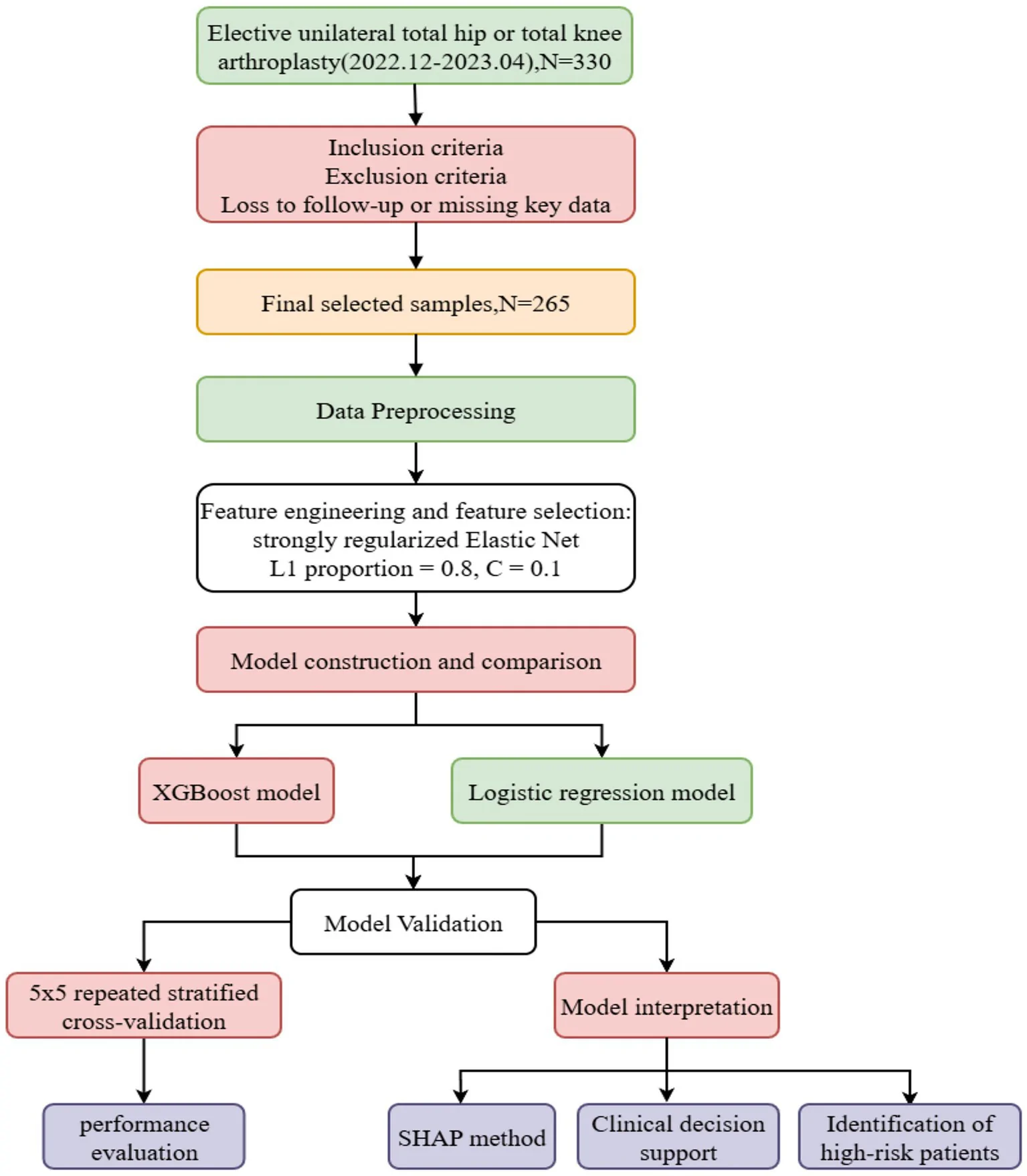
Technical route map of this study.

This study employed a retrospective observational cohort design to construct and validate a risk prediction model for poor discharge readiness based on clinical and psychosocial factors. The sample size was calculated based on the predictive performance requirements of a binary logistic regression model. A significance level of *α* = 0.05 (two-sided) and a power of (1 − *β*) = 80% were set. The expected model AUC was ≥0.75, and the incidence of the outcome variable (poor discharge readiness) was approximately 50%. According to the empirical rule proposed by Vittinghoff et al. of “10 outcome events per predictor variable,” and considering the approximately 15 candidate variables retained after the initial screening in this study, the minimum number of events required was 150, and the total sample size should be at least 300. Considering an estimated 10% loss to follow-up or missing key data, the final target sample size was determined to be 330. A total of 265 cases were actually included in the analysis, which was slightly lower than the planned sample size. However, a *post hoc* power analysis indicated that the statistical power was still sufficient to meet the primary modeling and comparison requirements. Figure 1 shows the technical route of this study.

### 2.2 Study design and patients

The study was conducted in the orthopedic ward of the Affiliated Hospital of Putian University, a tertiary grade A hospital in Fujian Province, from December 2022 to April 2023. Adult patients undergoing elective unilateral lower extremity joint replacement surgery (total hip arthroplasty or total knee arthroplasty) were recruited using consecutive sampling. The study protocol was approved by the Ethics Committee of the Affiliated Hospital of Putian University (Approval No.: 2022083), and all participants provided written informed consent.

Inclusion criteria: (1) Age ≥18 years; (2) First-time unilateral hip or knee arthroplasty; (3) Conscious, with basic reading and writing skills, and able to complete the questionnaire independently or with the assistance of the researcher; (4) Voluntary participation and signing of the informed consent form.

Exclusion criteria: (1) Severe cognitive impairment, mental illness or communication disorder; (2) Emergency surgery, simultaneous bilateral joint replacement or revision surgery; (3) Severe postoperative complications (such as deep vein thrombosis, infection, cardiovascular and cerebrovascular events) requiring transfer to the ICU; (4) Withdrawal from the study midway or a missing rate of key variables >20%.

### 2.3 Data collection

Upon thorough examination, no missing values were identified across any of the 15 study variables for all 265 participants (100% completeness rate). The pre-specified data management protocol stipulated that for questionnaires with missing items ≤20%, the median (for continuous variables) or mode (for categorical variables) would be used for imputation, while questionnaires with missing items > 20% would be excluded as invalid. However, due to rigorous on-site quality control during data collection, including real-time verification of questionnaire completeness by trained research nurses prior to patient discharge, all questionnaires were returned fully completed, and no imputation was required. A summary of data completeness is provided in Supplementary Table S1.

### 2.4 Data preprocessing

The Tampa Scale for Kinesiophobia (TSK-13) was administered at two time points: within 24 h preoperatively (TSK_pre) and within 24 h before discharge (TSK_post). The total score for each time point was calculated at the scale level (i.e., the sum of all item responses). The derived variable TSK_change was computed as TSK_pre minus TSK_post. As no missing values were present in the dataset, no imputation was performed for any variable. The previously described imputation strategy represents a pre-specified contingency protocol that was not applied.

## 3 Measurement tools

### 3.1 General information questionnaire

This questionnaire was designed by the research team based on the opinions of clinical experts, covering the demographic and disease-related variables mentioned above. The content validity was evaluated by three orthopedic nursing experts with associate senior professional titles or above (Content Validity Index, CVI = 0.92), confirming that the items were representative and applicable.

### 3.2 Tampa scale for kinesiophobia-13 (TSK-13)

This scale consists of 13 items (15) and uses a 4-point Likert scale (1 = strongly disagree, 2 = disagree, 3 = agree, 4 = strongly agree). The TSK-13 is typically composed of 13 positively scored items from the TSK-17 scale, excluding the 4 negatively scored items. The total score ranges from 13 to 52, with a total score of ≥23 indicating the presence of clinically significant kinesiophobia (15). This scale has good psychometric properties in patients with musculoskeletal pain. In orthopedic postoperative populations (such as patients with shoulder instability), its internal consistency (Cronbach’s *α* coefficient) is 0.851, and its test–retest reliability (intraclass correlation coefficient ICC) is 0.9151. In patients with chronic pain, its ICC also shows a good to excellent level (ICC = 0.88) (16).

### 3.3 Readiness for hospital discharge scale (RHDS)

This scale consists of 12 items (17), covering three dimensions: personal status (such as pain control, physical recovery), adaptability (such as self-care skills, activity ability), and expected support (such as family support, community resources). Its assessment is crucial for facilitating a safe transition to the home environment, especially for patients who have undergone severe physical trauma or complex surgeries. Each item is scored on a 0–10 visual analogue scale (0 = completely unprepared, 10 = completely prepared), with a total score ranging from 0 to 120. The scale demonstrates excellent psychometric properties, with a content validity index (CVI) of 0.88 and a Cronbach’s *α* coefficient of 0.89, indicating reliable internal consistency and structural validity.

### 3.4 Data collection methods

Two research nurses who received unified training were responsible for data collection. A standardized operation procedure was adopted to minimize information bias as much as possible:

Baseline demographic and clinical data: Within 24 h of admission, the following information was collected through electronic medical record review and face-to-face interviews using a self-designed general information questionnaire: age (years), gender (male/female), educational level (primary school or below, junior high school, high school/technical secondary school, junior college/bachelor’s degree or above), marital status (married, unmarried, divorced/widowed), occupation (farmer or worker, ordinary employee, professional and technical personnel, unemployed, other), monthly family income (<3,000 yuan, 3,000–5,000 yuan, 5,000–10,000 yuan, >10,000 yuan), place of residence (urban, rural), number of underlying diseases (none, one, two or more), type of surgery (total hip arthroplasty, total knee arthroplasty), payment type (fully self-paid, urban medical insurance, public medical care), inpatient caregiver (children, spouse, nurse, other), and length of hospital stay (days).

Kinesiophobia assessment: Within 24 h before the operation, the research nurse guided the patients one-on-one to complete the Chinese version of the TSK-13 scale.

Re-assessment of kinesiophobia after surgery: The TSK-13 was re-administered within 24 h before discharge to calculate the change value of kinesiophobia (TSK_change).

Discharge readiness assessment: On the morning of discharge, after morning care, the patients independently completed the Chinese version of the RHDS scale in a quiet environment.

All questionnaires were collected on the spot and checked for completeness by a second researcher. For questionnaires with missing items ≤20%, the median (for continuous variables) or mode (for categorical variables) was used to fill in the missing values; questionnaires with missing items >20% were considered invalid and excluded.

### 3.5 Machine learning modeling

To meet the requirements of machine learning modeling, in this study, the total score of RHDS was input into the model as a continuous variable, and the median of its sample (97.0 points) was used as the critical value for binary classification. The dependent variable was defined as: y = 1 (RHDS < 97, PDP), y = 0 (RHDS ≥97, Good Discharge Preparation, GDP).

The derived variable TSK change was created. For continuous variables, missing values were imputed with the median and then standardized using Z-score. For categorical variables, the mode was used for imputation followed by one-hot encoding (One-Hot Encoding, with the first category as the reference). A strongly regularized Elastic Net logistic regression (l1 ratio = 0.8, regularization strength C = 0.1) was applied to all candidate variables, and only those with non-zero coefficients were retained for subsequent modeling to enhance model interpretability and prevent overfitting. XGBoost gradient boosting tree models and Elastic Net logistic regression models were established, respectively. A strict evaluation was conducted using 5 × 5 repeated stratified K-fold cross-validation (Repeated Stratified K-Fold Cross-Validation) to ensure that the proportion of the two types of samples was consistent in each fold. The out-of-fold prediction results were used to calculate the following metrics: (1) Discrimination: area under the receiver operating characteristic curve (AUC), area under the precision-recall curve (PR-AUC).

Classification performance: sensitivity, specificity, F1 score; (2) Calibration: calibration curve (Calibration Curve);

Clinical utility: decision curve analysis (Decision Curve Analysis, DCA). Meanwhile, the 95% CI of AUC and PR-AUC were calculated by the Bootstrap method (repeated sampling 1,000 times). The DeLong test was used to compare the statistical significance of the AUC difference between the two models (*p* < 0.05). For the XGBoost model, the SHAP (SHapley Additive exPlanations) method was adopted to quantify the contribution of each feature to individual predictions, generating SHAP summary plots, dependence plots, and waterfall plots to reveal key predictive factors and their directions of effect.

Data analysis was conducted using Python 3.10 (scikit-learn, XGBoost, SHAP libraries). Descriptive statistics: Continuous variables were found to be non-normally distributed by Shapiro–Wilk test and Q-Q plot, and were expressed as median [interquartile range (IQR)], with intergroup comparisons performed using the Mann–Whitney *U* test; categorical variables were expressed as frequency (%), and intergroup comparisons were conducted using the chi-square test or Fisher’s exact test (when the expected frequency was <5).

## 4 Results

### 4.1 Baseline characteristics and intergroup differences of the study population

A total of 265 patients undergoing elective unilateral lower extremity joint replacement surgery were ultimately included in this study. Based on the median total score of the RHDS of 97.0, 117 cases (44.2%) were classified as the PDP group, and 148 cases (55.8%) as the GDP group. As shown in Table 1, the two groups were evenly distributed in terms of demographic variables such as gender, education level, marital status, occupation, number of underlying diseases, payment method, caregiver, and type of surgery (all *p* > 0.05). However,patients in the poor discharge preparation group were significantly more likely to come from low-income families (monthly household income <3,000 RMB: 41.0% vs. 21.6%, *p* = 0.001) and were more likely to reside in urban areas (23.9% vs. 8.8%, *p* = 0.003), and had a significantly shorter hospital stay (median 10 days [IQR: 9–11] vs. 14 days [IQR: 11–18], *p* < 0.001). In terms of psychological indicators, the poor discharge preparation group had a higher preoperative fear of movement level (TSK_pre) (median 36 [33–39] vs. 34 [32–37], *p* = 0.009), and the postoperative fear of movement (TSK_post) did not fully alleviate (30 ^[28–33]^ vs. 28[27–30], *p* < 0.001), while the difference in the change value of fear of movement (TSK_change) between the groups was not statistically significant (*p* = 0.358). These findings suggest that socioeconomic status and hospital stay length may be key non-clinical factors affecting discharge readiness.

**Table 1:** Clinical characteristics of patients undergoing lower extremity joint replacement (*N* = 265).

Variable	Good discharge preparation (*N* = 148)	Poor discharge preparation (*N* = 117)	*p*-value
Age	65.00 [50.00, 65.00]	65.00 [50.00, 65.00]	0.202
Gender			1
Female	83 (56.1%)	66 (56.4%)	
Male	65 (43.9%)	51 (43.6%)	
Underlying_Disease			0.23
0	49 (33.1%)	44 (37.6%)	
1	48 (32.4%)	44 (37.6%)	
>1	51 (34.5%)	29 (24.8%)	
Payment_Type			0.438
Fully self-financed	0 (0.0%)	1 (0.9%)	
Public medical care	16 (10.8%)	13 (11.1%)	
Urban medical insurance	132 (89.2%)	103 (88.0%)	
Hospital_Caregiver			0.123
Caregiver	4 (2.7%)	2 (1.7%)	
Offspring	62 (41.9%)	64 (54.7%)	
Other	5 (3.4%)	1 (0.9%)	
Spouse	77 (52.0%)	50 (42.7%)	
Education_Level			0.383
Associate/bachelor’s degrees	13 (8.8%)	8 (6.8%)	
Lower secondary education	45 (30.4%)	41 (35.0%)	
Primary schools and below	62 (41.9%)	39 (33.3%)	
Vocational/general secondary education	28 (18.9%)	29 (24.8%)	
Marital_Status			0.468
Married	142 (95.9%)	109 (93.2%)	
Single	6 (4.1%)	8 (6.8%)	
Monthly_Household_Income(RMB)			0.001
3,000–5,000	60 (40.5%)	26 (22.2%)	
5,001–10,000	40 (27.0%)	33 (28.2%)	
<3,000	32 (21.6%)	48 (41.0%)	
>10,000	16 (10.8%)	10 (8.5%)	
Residence			0.003
City	13 (8.8%)	28 (23.9%)	
Rural	62 (41.9%)	39 (33.3%)	
Town	73 (49.3%)	50 (42.7%)	
Occupation			0.971
Farmer/Worker	51 (34.5%)	43 (36.8%)	
Other	16 (10.8%)	12 (10.3%)	
Staff	32 (21.6%)	26 (22.2%)	
Technical personnel	34 (23.0%)	23 (19.7%)	
Unemployed	15 (10.1%)	13 (11.1%)	
Surgery_Type			0.91
THA	73 (49.3%)	56 (47.9%)	
TKA	75 (50.7%)	61 (52.1%)	
Days_Hospitalized	14.00 [11.00, 18.00]	10.00 [9.00, 11.00]	<0.001
TSK_pre	34.00 [32.00, 37.00]	36.00 [33.00, 39.00]	0.009
TSK_post	28.00 [27.00, 30.00]	30.00 [28.00, 33.00]	<0.001
TSK_change	6.00 [4.00, 8.00]	5.00 [3.00, 8.00]	0.358
RHDS	103.00 [99.00, 108.00]	92.00 [86.00, 95.00]	<0.001

THA, Total Hip Arthroplasty; TKA, Total Knee Arthroplasty; RHDS, Readiness for Hospital Discharge Scale; TSK-13, Tampa Scale for Kinesiophobia-13.

### 4.2 Prediction model construction and cross-validation performance

**Figure 2:**
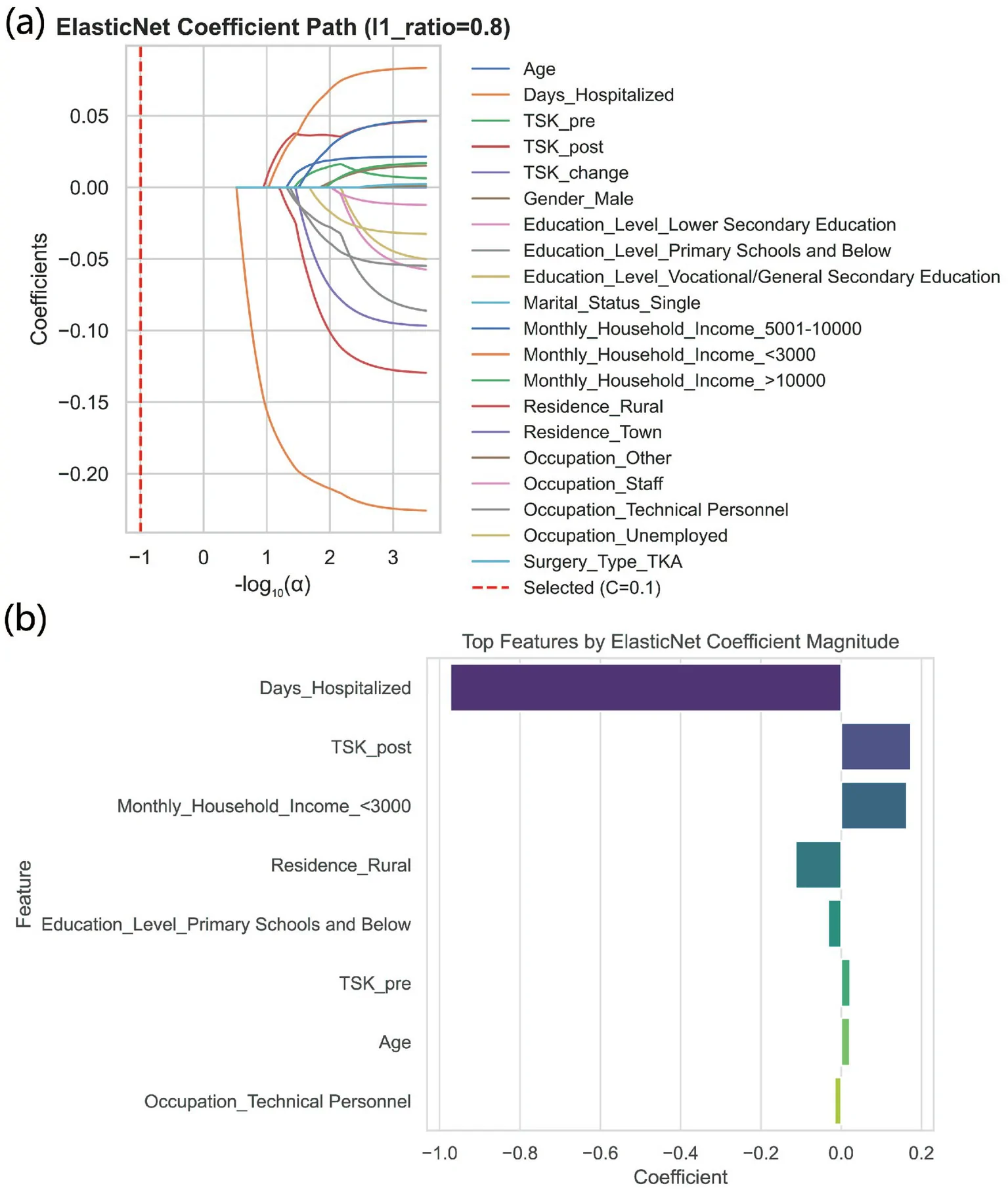
Screening and ranking of predictive factors. **(a)** Eight non-zero coefficient predictive factors retained after variable screening based on Elastic Net logistic regression (l1_ratio = 0.8, C = 0.1). **(b)** Bar chart of the importance of the selected predictive factors ranked by the absolute value of their regression coefficients.

To identify high-risk individuals, we constructed a prediction model based on 25 original variables (including the derived variable TSK_change). Variable selection was performed using strongly regularized Elastic Net logistic regression (l1_ratio = 0.8, C = 0.1), and ultimately, 8 predictors with non-zero coefficients were retained (Figure 2a). These factors were ranked in order of importance based on their coefficients as follows: length of hospital stay, postoperative TSK, monthly household income, educational level, preoperative TSK, age, and occupation (Figure 2b).

**Figure 3:**
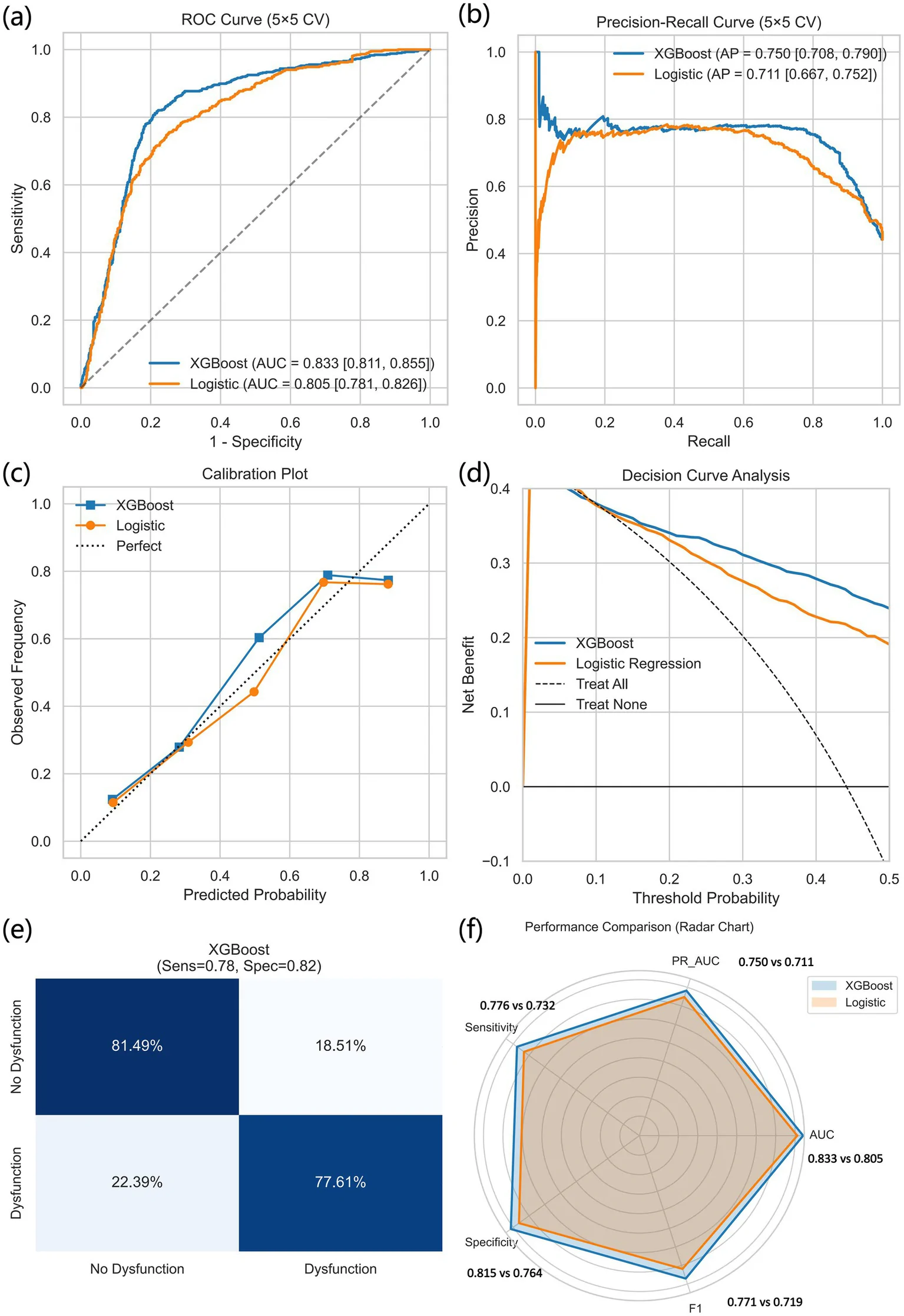
Model performance comparison and clinical decision-making value. **(a,b)** Receiver operating characteristic curves **(a)** and precision-recall curves **(b)** of XGBoost and Elastic Net logistic regression under 5 × 5 repeated stratified cross-validation. The shaded areas represent the 95% confidence intervals. **(c)** Calibration curves of the two models, with the diagonal line indicating ideal calibration. **(d)** Decision curve analysis, showing the net benefit of each strategy within the threshold probability range of 5–40%. **(e)** Confusion matrix of the XGBoost model on the test set. **(f)** Comprehensive performance comparison of the two models in terms of AUC, PR-AUC, and F1 score.

Based on this, we trained and compared two models, XGBoost and Elastic Net logistic regression, and evaluated their generalization ability using a strict 5 × 5 repeated stratified cross-validation. The XGBoost model demonstrated excellent discriminatory performance (Figures 3a,b): its AUC was 0.833 (95% CI: 0.811–0.855), and its PR-AUC was 0.750 (95% CI: 0.708–0.790). In contrast, the performance of the logistic regression model was slightly inferior (AUC = 0.805, 95% CI: 0.781–0.826; PR-AUC = 0.711, 95% CI: 0.667–0.752). The DeLong test confirmed that the AUC advantage of XGBoost was statistically significant (*p* = 0.0035). Calibration curves indicated that the predicted probabilities of both models were highly consistent with the actual observed frequencies, confirming their good calibration (Figure 3c).

### 4.3 Visualization of clinical decision value and comprehensive performance

To evaluate the practical application value of the model, we conducted a decision curve analysis. It indicated that within the clinically relevant threshold probability range (5–40%), the XGBoost model consistently provided the highest net benefit, significantly outperforming the logistic regression model, the default strategies of “intervene all” or “intervene none,” demonstrating its clear clinical utility (Figure 3d). The confusion matrix further quantified the classification performance: XGBoost achieved a recognition rate of 77.6% for patients with poor discharge readiness and correctly excluded 81.5% of low-risk patients (Figure 3e). Additionally, in terms of the F1 score, the XGBoost model also outperformed logistic regression (0.771 vs. 0.719). These results collectively confirmed the comprehensive advantages of XGBoost (Figure 3f).

### 4.4 Model interpretability and mechanism of key predictors

**Figure 4:**
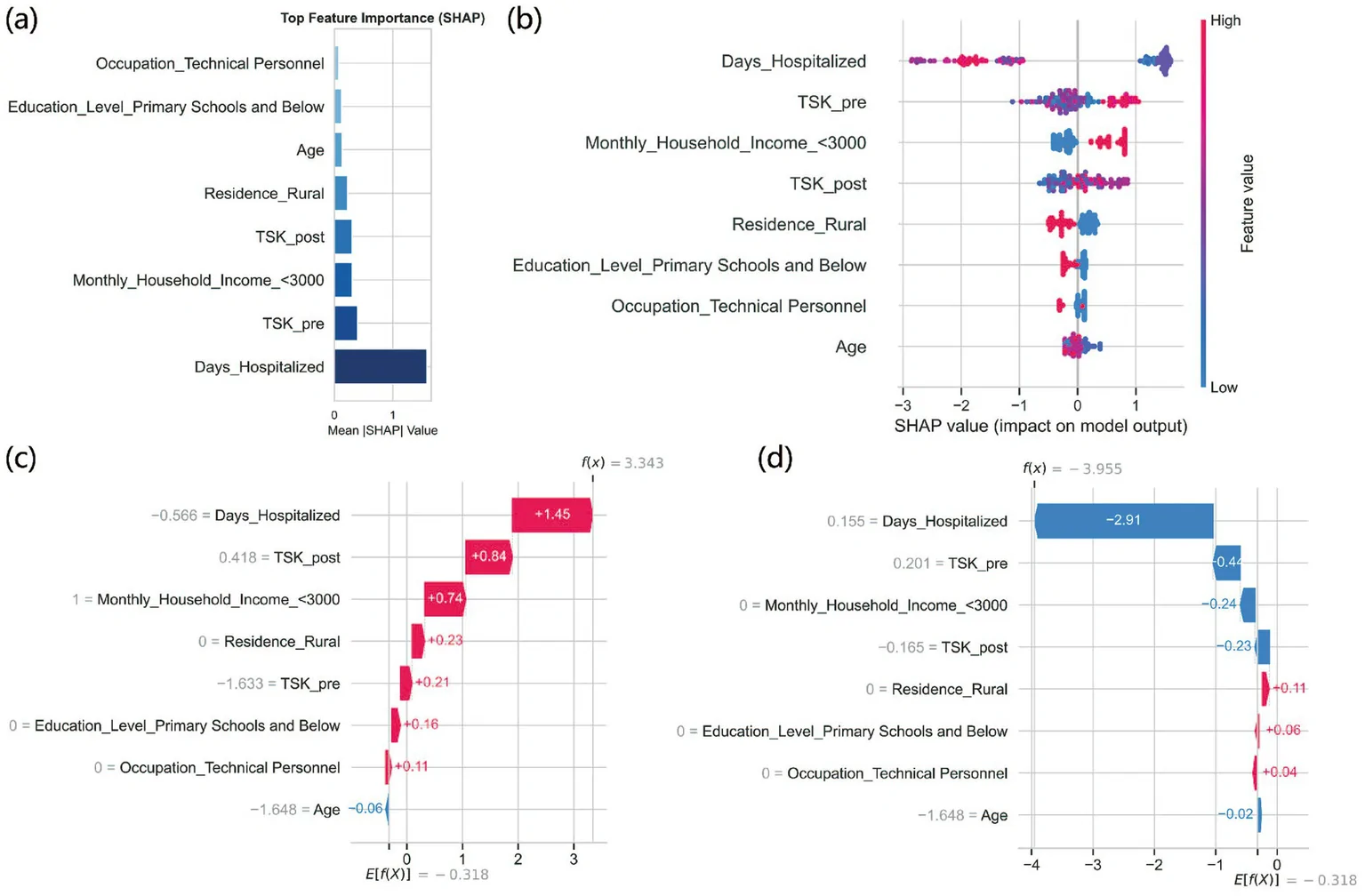
SHAP explainability analysis of the XGBoost model. **(a)** Feature importance ranking based on the average |SHAP| value. **(b)** SHAP beeswarm plot, demonstrating the direction and influence degree of key features on the prediction results. **(c)** SHAP waterfall plot of a high-risk individual, showing the main risk-driving features and their contributions. **(d)** SHAP waterfall plot of a low-risk individual, illustrating the role of protective features.

To reveal the decision-making logic of the model, we used the SHAP method to post-hoc explain the XGBoost model. Feature importance analysis showed that the length of hospital stay was the primary predictor (Mean |SHAP| = 1.583), followed by preoperative fear of movement (TSK_pre, 0.394), low family income (<3,000 yuan, 0.303), and postoperative fear of movement (TSK_post, 0.301, Figure 4a). The SHAP beeswarm plot revealed the patterns of key variables: the shorter the hospital stay, the higher the risk of poor discharge readiness; higher preoperative fear of movement (TSK_pre) levels, low-income status, being a professional technician, living in rural areas, having an education level of primary school or below, and older age were all associated with a greater risk of poor discharge readiness (Figure 4b). For instance, the predicted outcome for a high-risk patient is mainly driven by a higher level of postoperative activity fear (TSK_post +0.84), lower income (+0.74), and an exceptionally long hospital stay (+1.45) (Figure 4c). In contrast, the protective characteristics for a low-risk patient are reflected in the TSK_pre value and a shorter hospital stay (Figure 4d).

## 5 Discussion

This study developed a predictive model for poor discharge readiness risk in patients undergoing elective unilateral lower extremity joint replacement based on clinical indicators and psychosocial factors, aiming to identify high-risk patients early and guide personalized interventions. By retrospectively collecting multi-dimensional data of patients and combining modern statistical and machine learning methods, the key factors influencing discharge readiness were systematically screened out, revealing the interaction between clinical status and psychosocial background. The results showed that the model had good discrimination, and identified short hospital stay, high preoperative kinesiophobia level, and low family monthly income as core predictive factors. The following text will delve into the clinical significance of these key findings, their similarities and differences with existing evidence, and the potential value of model application.

This study reveals that the length of hospital stay, preoperative kinesiophobia level, family income, and educational attainment are key risk factors for poor discharge readiness, suggesting that the complex interaction mechanisms of these variables in the patient’s rehabilitation process deserve in-depth exploration. From both physiological and psychological perspectives, short hospital stays may prevent patients from completing necessary functional recovery and psychological adaptation, increasing the risk of postoperative complications and readmission. Previous studies have indicated that the length of hospital stay is closely related to postoperative inflammatory responses and tissue repair processes. Early discharge may leave patients in a state of low immune function and incomplete tissue repair, hindering recovery (18). Additionally, preoperative kinesiophobia, as a psychological state, may promote pain amplification and movement avoidance, activating the hypothalamic–pituitary–adrenal axis, triggering chronic stress responses and increasing the release of inflammatory mediators, thereby delaying tissue healing and functional recovery (19). The influence of family income and educational attainment may be mediated by social determinants, such as economic pressure limiting patients’ access to rehabilitation resources, and lower educational levels leading to insufficient health literacy, affecting patients’ self-management and compliance (20, 21). This study combines Elastic Net, logistic regression, and XGBoost models to precisely quantify the effects of these multi-dimensional factors, providing a new perspective on how socioeconomic and psychological factors interact to influence discharge readiness.

Preoperative kinesiophobia was significantly associated with poor discharge readiness in patients, suggesting the potential value of psychological intervention in preoperative rehabilitation plans. Kinesiophobia enhances patients’ attention to pain and negative cognition, activates the stress response of the central nervous system, leading to excessive excitation of the sympathetic nervous system and elevated inflammatory factors, which affect the healing process and functional recovery (18, 19). Contrary to traditional views, this study emphasizes that kinesiophobia not only influences patients’ behavioral avoidance of movement but also regulates tissue repair and immune responses through neuroendocrine pathways, revealing the interweaving of psychological states and biological mechanisms. In existing literature, cognitive behavioral therapy for kinesiophobia has shown preliminary effects in alleviating pain and improving function, but most scholars point out the lack of high-quality randomized controlled trials to verify its postoperative effects (19). This study clearly identified kinesiophobia as an important indicator of preoperative psychological burden, suggesting that future research should deepen the exploration of the neuroimmune mechanisms mediated by kinesiophobia and combine individualized psychological intervention strategies to optimize preoperative preparation and rehabilitation pathways.

Socioeconomic status influences patients’ discharge readiness through multiple mechanisms, specifically reflected in the moderating effects of family income and educational attainment on access to medical resources and health behaviors. Lower family income restricts patients’ ability to obtain high-quality postoperative rehabilitation support and assistive devices, while increasing life stress and psychological burden, which may lead to reduced compliance with rehabilitation plans (20). Patients with lower educational levels often lack the necessary knowledge for disease management and have difficulty effectively implementing postoperative self-care and rehabilitation guidance (21). Additionally, socioeconomic factors also affect patients’ social support networks, further influencing their psychological state and rehabilitation motivation (22). Compared with previous studies that focused solely on clinical factors, this study comprehensively considers the interaction between socioeconomic indicators and psychological variables, emphasizing the core role of social determinants in postoperative rehabilitation and promoting the development of discharge readiness assessment towards a multi-dimensional and personalized direction.

The superior performance of machine learning models in predicting discharge readiness risk, particularly the XGBoost model which significantly outperforms the traditional Elastic Net logistic regression, reflects the importance of nonlinear variable interactions and high-dimensional data structures in clinical risk assessment. XGBoost, through the ensemble method of gradient boosting decision trees, effectively captures complex nonlinear relationships and interaction effects among variables, overcoming issues of multicollinearity and variable redundancy (23). Compared with traditional statistical models, machine learning can adaptively mine potential features without the need to pre-assume data distribution, enhancing the accuracy and generalization ability of predictions (24). However, the “black box” nature of machine learning models in clinical applications limits their interpretability. In recent years, model-agnostic explanation tools have emerged, providing a solution by offering local explanations and global feature importance analysis to make the prediction paths of complex models transparent (25). This study innovatively combines multiple machine learning techniques for variable selection and model validation in methodology, enhancing the clinical interpretability and practicality of the model, and laying the foundation for the future development of personalized risk assessment tools integrating clinical and psychosocial factors.

Decision curve analysis demonstrated that the XGBoost model provided higher net benefits within the threshold probability range of 5 to 40%, validating its practicality in clinical decision-making. This model can assist doctors in identifying high-risk patients, enabling early intervention and reducing postoperative complications and readmission rates (26). Compared with traditional experience-based assessment methods, machine learning models optimize the decision-making process through data-driven approaches, reducing subjective bias and improving resource allocation efficiency (27). While validating the clinical value of the model, this study also proposed strategies for its promotion and application, providing a practical foundation for future multi-center promotion and cross-regional applicability research.

To the best of our knowledge, no previous study has developed a machine-learning model specifically to predict poor discharge readiness in patients undergoing unilateral lower extremity joint replacement. Existing predictive studies in arthroplasty have primarily focused on outcomes such as readmission, length of stay, discharge destination, or functional recovery, whereas studies involving the RHDS have generally been descriptive or association-based rather than predictive. Therefore, the present study extends the literature by focusing specifically on discharge readiness as the primary outcome and by combining predictive modeling with SHAP-based interpretability.

It should be noted that the definition of poor discharge readiness using the sample median RHDS score (97.0) is a data-driven approach with inherent limitations. Currently, there is no universally accepted or clinically validated cutoff value for the RHDS in the context of joint replacement surgery. In the absence of an established clinical threshold, the sample median was used as it ensures balanced group sizes and avoids arbitrary threshold selection. However, this cutoff has not been validated against hard clinical endpoints such as delayed discharge, 30-day readmission, or postoperative functional recovery scores. Patients classified as having poor discharge readiness based on this cutoff may not necessarily experience adverse clinical outcomes, and vice versa. The data-driven nature of this cutoff also means it may not be directly applicable to other patient populations or clinical settings with different baseline characteristics. Future studies are recommended to establish clinically validated RHDS cutoff values linked to objective outcomes such as readmission rates and functional recovery measures.

Several limitations of this study should be acknowledged. First, this was a single-center study conducted in a tertiary hospital in Fujian Province, which may limit the generalizability of the findings to other regions or healthcare settings with different patient characteristics and clinical practices. Second, the final sample size (*n* = 265) was smaller than the initially planned target (*n* = 330), primarily because of the limited enrollment period and strict inclusion and exclusion criteria. Although the model showed robust performance and good calibration, the reduced sample size may have limited the ability to detect weaker predictors. Third, although internal validation was strengthened by 5 × 5 repeated stratified cross-validation and SHAP-based interpretability analysis, external validation in independent cohorts remains necessary before the model can be applied in routine clinical practice. Future multicenter studies with larger samples are needed to evaluate the model’s transportability and generalizability. Fourth, poor discharge readiness was defined using a data-driven cutoff based on the sample median RHDS score (97 points), rather than a threshold validated against objective clinical outcomes such as readmission or delayed functional recovery. This may reduce the clinical interpretability of the model outcome. Fifth, the predictors included in the model were mainly derived from self-reported scales and limited medical record data, without incorporating more comprehensive contextual variables, such as the quality of social support or the home rehabilitation environment. Therefore, some relevant determinants of discharge readiness may not have been fully captured.

Although the XGBoost model demonstrated strong predictive performance, its practical bedside application is currently limited by the lack of a simplified, user-friendly clinical tool. Machine learning ensemble models such as XGBoost are inherently complex and not easily reduced to simple scoring systems without some loss of predictive accuracy. To enhance clinical translatability, future work should focus on developing simplified clinical tools based on the eight key predictors identified in this study, including: (1) a nomogram providing a visual and easy-to-use bedside risk estimation tool; (2) a risk stratification table categorizing patients into low, moderate, and high-risk groups based on SHAP-derived scores to facilitate clinical decision-making and targeted interventions; and (3) a web-based calculator enabling real-time risk assessment without requiring specialized software. These tools would bridge the gap between model development and clinical implementation.

## 6 Conclusion

This study successfully constructed and validated an interpretable machine learning prediction model integrating clinical and psychosocial factors, with particular attention to dynamic psychological indicators and modifiable clinical variables. The XGBoost model outperformed traditional logistic regression in identifying the risk of poor discharge readiness in patients after joint replacement surgery, demonstrating good discrimination, calibration, and clinical net benefit. These findings provide key targets for implementing precise and proactive nursing interventions. Future research should validate this model in multi-center, larger-sample studies and explore its integration into clinical decision support systems to enable real-time risk assessment, personalized discharge planning, and optimized allocation of medical resources.

## Data Availability

The raw data supporting the conclusions of this article will be made available by the authors, without undue reservation.
